# Effect of antimalarial prophylaxis with sulphadoxine-pyrimethamine on pregnancy outcomes in Nsukka, Nigeria

**DOI:** 10.5281/zenodo.10757166

**Published:** 2017-03-05

**Authors:** Nneka U. Igboeli, Chinwe V. Ukwe, Cletus N. Aguwa

**Affiliations:** 1 Department of Clinical Pharmacy and Pharmacy Management, Faculty of Pharmaceutical Sciences, University of Nigeria, Nsukka 410001, Nigeria

## Abstract

**Background:**

We evaluated the association between the use of intermittent preventive treatment with sulphadoxine-pyrimethamine (IPTp-SP) on pregnancy outcomes among women who delivered at a secondary hospital in Nsukka, Enugu State, Nigeria.

**Materials and methods:**

Relevant obstetric data (e.g. IPTp-SP use), matched against pregnancy outcome data such as delivery method, stillbirth, maternal haematocrit test results and babies’ birth weights, were collected retrospectively from antenatal care (ANC) case files of women who delivered within a one-year period (2013).

**Results:**

The prevalence of adverse pregnancy outcomes recorded out of the 500 ANC case files analysed were: low birth weight (LBW) 3.6% (15), anaemia 54.3% (114), caesarean section 31.6% (156) and stillbirth 3.6% (67). A total of 342 (68.4%) of the women received IPTp-SP during ANC and the receipt of IPTp-SP was significantly associated with reductions in the following events: LBW [OR = 0.26, 95% CI = 0.09 – 0.75], moderate anaemia [OR = 0.33, 95% CI = 0.17 – 0.63], caesarean section [OR = 0.36, 95% CI = 0.24 – 0.53] and stillbirth [OR = 0.10, 95% CI = 0.06 – 0.18].

**Conclusion:**

In this area of high malaria transmission we demonstrated significant reductions in unfavourable maternal and infant health outcomes when using IPT-SP.

## 1 Introduction

Malaria is frequently associated with adverse pregnancy outcomes for both the mother and newborn, particularly in high malaria transmission settings like Nigeria. These adverse outcomes include maternal morbidity, low birth weight, preterm delivery and perinatal mortality (stillbirth). The prevention and control of malaria in pregnancy is therefore necessary to reduce the morbidity and mortality associated with malaria for both the mothers and their children. Infected pregnant women in malaria-endemic regions are often asymptomatic and therefore require a preventive approach. The principal effects of malaria infection in pregnancy are malaria-related anaemia in the mother and the presence of parasites in the placenta [1]. The resultant impairment of foetal nutrition contributes to low birth weight (LBW), which is a leading cause of infant mortality [2]. These adverse events are highly prevalent in first pregnancies because of the increased susceptibility of primigravidae to infection with *Plasmodium falciparum* – the primary plasmodium species accounting for more than 90% of malaria infections in Nigeria.

Intermittent preventive treatment of malaria during pregnancy (IPTp) is a key intervention in the national strategy for malaria control in Nigeria. Sulphadoxine-Pyrimethamine (SP), the current drug of choice, is recommended to be administered in the second and third trimesters of pregnancies by directly-observed therapy, during antenatal care (ANC) visits. Although the World Health Organization (WHO) currently recommends monthly SP administration to pregnant women until delivery [3], the Nigerian policy still advocates the old policy of at least two doses in human immunodeficiency virus (HIV) negative pregnant women and 3 doses for HIV-positive pregnant women [4]. IPTp-SP has proven to be beneficial, with multiple studies demonstrating decreased maternal anaemia, decreased placental malaria, and increased birth weight [5]. We therefore aimed to evaluate the association between the use of IPTp-SP and pregnancy outcomes in women who delivered at the secondary hospital in Nsukka, Enugu State, Nigeria.

## 2 Materials and methods

This retrospective survey was conducted at a secondary hospital in Nsukka, Enugu state, Nigeria. The hospital is a mission hospital with 150 beds and an estimated average monthly delivery of 40. A convenience sample of all available and complete antenatal care (ANC) folders of women who delivered in 2013 were used. Relevant obstetric data (e.g. IPTp-SP use) matched against pregnancy outcome data such as delivery method, baby’s birth weights, stillbirth, maternal haemoglobin, haematocrit and microscopic malaria test results were collected retrospectively from the ANC folders. This study was carried out with the ethical clearance from University of Nigeria Teaching Hospital, Enugu and approval by the local hospital administration. The data were entered into excel and later exported to SPSS version 16 for data analysis. Low birth weight (LBW) was defined as < 2.5kg and maternal anaemia as haemoglobin (Hb) < 11g/dl. Descriptive statistics, mean, standard deviation and univariate logistic re-gression analyses were used to analyse the data where applicable. Significance was set at p < 0.05 for all analyses.

## 3 Results

### 3.1 Delivery cohort characteristics

A total of 500 ANC case files of women who delivered in 2013 were available for analysis. One-third, 140 (31.1%) of the women that delivered at the hospital during that period, were primigravids. The majority of these women, 261 (73.9%), had up to 4 antenatal care visits. During their prenatal period, the prevalence of anaemia [haemoglobin concentration < 11g/dl] in all women was high, 114 (54.3%), and also more than half of them tested positive for malaria [24 (55.8%)] among those that underwent blood tests (n = 43). The major route of birth was vaginal delivery 337 (68.4%). Slightly more than half of the babies were male 259 (54.9%). Only 67 (13.6%) stillbirths were recorded (Table 1).

**Table 1.** Characteristics of the delivery cohorts that received or not received IPTp-SP during pregnancy.

Variable	IPTp-SP used (%)	IPTp-SP not used (%)	n (%)
**Gravidity (n=450)**			
Primigravidae	68 (29.7)	38 (31.7)	136 (30.2)
Multigravidae	232 (70.3)	82 (68.3)	314 (69.8)
**Birth weight (n=416)**			
Low	7 (2.2)	8 (7.9)	15 (3.6)
Normal	308 (97.8)	93 (92.1)	401 (96.4)
# of ANC* visits (n=353)			
< 4	65 (21.9)	27 (48.2)	92 (26.1)
≥ 4	232 (78.1)	29 (51.8)	261 (73.9)
**Anaemia (n=210)**			
Hb** < 11g/dl	69 (46.6)	45 (72.6)	114 (54.3)
Hb ≥ 11g/dl	79 (53.4)	17 (27.4)	96 (45.7)
**Malaria (n=43)**			
Positive	16 (63.2)	8 (53.3)	24 (55.8)
Negative	12 (63.2)	7 (46.7)	19 (44.2)
**Mode of delivery (n=493)**			
Vaginal	256 (75.7)	82 (52.6)	337 (68.4)
Caesarean section	82 (24.3)	74 (47.4)	156 (31.6)
**Baby gender (n=472)**			
Female	155 (46.1)	58 (42.6)	213 (45.1)
Male	181 (53.9)	78 (57.4)	259 (54.9)
**Baby condition (n=492)**			
Alive	321 (95.0)	103 (66.9)	424 (86.2)
Stillbirth	16 (4.7)	51 (33.1)	67 (13.6)
Intra-uterine foetal death	1 (0.3)	0 (0.0)	1 (0.2)

*= Antenatal care; **= Haemoglobin

### 3.2 Prevalence and risks of adverse pregnancy outcomes

Prevalence and risks associated with adverse pregnancy outcomes among women that received IPTp-SP compared to those that did not receive any SP dose for IPTp are shown in Table 2. The prevalence of LBW deliveries among those who did not receive any IPTp-SP was three times lower than the prevalence in those that did received the treatment (7.9% vs. 2.2%). Thus, use of IPTp-SP was associated with a significant risk reduction of having LBW [OR = 0.26, 95% CI = 0.09-0.75, p = 0.012]. Also, the prevalence rates of having anaemia (72.6% vs. 46.6%), delivering through caesarean section (47.4% vs. 24.3%) and having a stillbirth (33.1% vs. 4.7%) were all significantly higher among women who did not receive any IPTp -SP when compared with those who received IPTp-SP. These led to corresponding risk reductions of these outcomes among women that received IPTp-SP: anaemia [OR = 0.33, 95% CI = 0.17-0.63, p = 0.001], caesarean section [OR = 0.36, 95% CI = 0.25-0.53, p < 0.001] and stillbirth [OR = 0.10, 95% CI = 0.06-0.18, p < 0.001].

**Table 2.** Association between the use of IPTp-SP and outcomes of pregnancy.

Variable	IPT-SP used (%)	IPT-SP not used (%)	Odds ratio (95% CI)	p
**Birth weight**				
Low	7 (2.2)	8 (7.9)	0.26 (0.09-0.75)	0.012
Normal	308 (97.8)	93 (92.1)		
**Anaemia**				
Hb < 11g/dl	69 (46.6)	45 (72.6)	0.33 (0.17-0.63)	0.001
Hb > 11g/dl	79 (53.4)	17 (27.4)		
**Delivery route**				
Vaginal delivery	256 (75.7)	82 (52.6)	0.36 (0.24-0.53)	<0.001
Caesarean section	82 (24.3)	74 (47.4)		
**Baby condition at birth**				
Alive	321 (95.0)	103 (66.9)	0.10 (0.06-0.18)	<0.001
Stillbirth	16 (4.7)	51 (33.1)		
Intra-uterine foetal death	1 (0.3)	0 (0.0)		

### 3.3 Effect of IPTp-SP receipt on birth weight and packed cell volume

The receipt of IPTp-SP significantly increased the baby’s birth weight by 200g [mean birth weight difference, kg = 0.20, 95% CI = 0.08-0.32, p < 0.001]. The mean packed cell volume among women that received IPTp-SP was also higher [mean packed cell volume difference, % = 4.05, 95% CI = 2.53-5.57, p < 0.001] (Table 3).

**Table 3.** Mean (±SD) and mean differences in birth weight and Packed Cell Volume (PCV) by use of IPTp-SP.

Variable	IPTp-SP used	IPTp-SP not used	Mean (95Difference % CI)	p
Birth weight, kg (Mean±SD)	3.41 ± 0.51	3.21 ± 0.59	0.20 (0.08-0.32)	<0.001
PCV, % (Mean±SD)	32.62 ± 4.48	28.58 ± 6.37	4.05 (2.53-5.57)	<0.001

## 4 Discussion

Adverse pregnancy outcomes were found to be significantly reduced in women that received IPTp-SP compared to those that did not. The risks of stillbirth and low birth weight were the outcomes most affected by the use of IPTp-SP.

The National Treatment Guideline recommends that each pregnant woman receives at least two IPTp-SP doses during ANC visits after quickening and these were based on reports of beneficial effects of IPTp-SP in preventing maternal malaria and improving pregnancy outcomes in studies conducted in Africa [6-8]. Despite these beneficial effects, implementation has been suboptimal as was recorded in this study and in national surveys [9,10].

In a meta-analysis of 32 national surveys, IPTp-SP was found to be beneficial, resulting in 17% reduction in low birth weight and 16% reduction in neonatal mortality [11]. It has been argued, however, that the result of the meta-analysis may have underestimated the benefit of reduced LBW associated with IPTp-SP and insecticide treated bed-nets (ITNs) due to the fact that reported, as opposed to measured birth weight, by mothers was used in the surveys. This study reported a reduction in the risk of LBW of 74% and the use of actual birth weight measurements may have contributed to this huge disparity in proportions of risk reduction. The effect of IPTp-SP on neonatal mortality has been ascribed to the increase in birth weight with IPTp-SP use [11]. Low birth weight due to prematurity or intra-uterine growth retardation caused predominantly by malaria has been identified as a significant risk factor for neonatal mortality [2,12]. However, a placebo-controlled trial of IPTp-SP found a marked reduction (61.3%) in neonatal mortality without an effect on birth weight, suggesting that the treatment affects neonatal survival through mechanism(s) independent of increased birth weight [13]. A huge reduction (90%) in stillbirth was recorded for those women that took IPTp-SP in the present study.

The cause of maternal anaemia is multifactorial, but it has been reported that the proportion of severe anaemia among women of all gravidities attributable to malaria (population attributable fraction) is estimated to be 26% [12]. The use of IPTp-SP has been shown to reduce the risk of maternal anaemia in Nigeria [14] as was also found in our study. In fact, a meta-analysis of antimalarial prophylaxis trials with intermittent preventive treatment suggests that successful prevention of malaria reduces the risk of severe anaemia by 38% (95% CI = 22-50%) [15]. However, a more substantial reduction in risk (67%) was reported in our study. Vaginal delivery is safer than caesarean section if there are no complications with the pregnancy. However, a caesarean section, particularly if it is planned, is a common and safe procedure. The risk of having caesarean section was significantly reduced among women that took SP in our study. Common indicators for caesarean section include failure of labour to progress (often due to cephalopelvic disproportion), eclampsia (including severe pre-eclampsia), breech presentation, foetal distress and antepartum haemorrhage [16-19]; most of these have no direct association with malaria. Preeclampsia [20] has been associated with malaria, but its contributions to the risk of caesarean section in this study were not evaluated. On the other hand, it has been reported that in developing countries like Nigeria, there is still a great aversion to caesarean section [21, 22] and due to the structuring of the Nigerian health system, majority of deliveries are initiated in centres where caesarean section cannot be offered. This most probably accounts for the high rates of unbooked patients seen in labour at the referral hospitals that often are more likely to undergo emergency caesarean section with adverse obstetric outcomes compared to booked parturients [23-25]. It is possible that, the study site being a referral hospital in that area, the majority of the caesarean sections were for unbooked women with no record of IPTp-SP use in their ANC folders, thereby giving a false positive reduction in the risk of caesarean section among booked parturient with records of IPTp-SP use. Another important confounder to this result is the possibility that women who received IPTp may have better access to care and thus might be less likely to need caesarean section. In as much as there is no direct association between malaria and risk of having caesarean section, this result should be investigated further in a prospective study with relevant data on possible confounders in order to ascertain this association. A major limitation in this study is that it is a retrospective study with non-randomisation of study participants. Thus, any of its findings could be due to unmeasured confounding variables. Nonetheless, these huge beneficial effects of IPTp-SP should not be overlooked notwithstanding the possibility of confounders.

## 5 Conclusions

In conclusion, although concerns have been raised about the effectiveness of SP in improving pregnancy outcomes (e.g. because of potential resistance), this study has demonstrated considerable reductions in adverse maternal and infant health outcomes with use of intermittent preventive treatment with SP in this area of high malaria transmission.
